# Canonical and Noncanonical Roles of Fanconi Anemia Proteins: Implications in Cancer Predisposition

**DOI:** 10.3390/cancers12092684

**Published:** 2020-09-20

**Authors:** Giacomo Milletti, Luisa Strocchio, Daria Pagliara, Katia Girardi, Roberto Carta, Angela Mastronuzzi, Franco Locatelli, Francesca Nazio

**Affiliations:** 1 Department of Pediatric Hemato-Oncology and Cell and Gene Therapy, Istituto di Ricovero e Cura a Carattere Scientifico Bambino Gesù Children’s Hospital, 00146 Rome, Italy; giacomo.milletti@opbg.net (G.M.); luisa.strocchio@opbg.net (L.S.); daria.pagliara@opbg.net (D.P.); katia.girardi@opbg.net (K.G.); roberto.carta@opbg.net (R.C.); angela.mastronuzzi@opbg.net (A.M.); franco.locatelli@opbg.net (F.L.); 2 Department of Gynecology/Obstetrics and Pediatrics, Sapienza University of Rome, 00161 Rome, Italy

**Keywords:** Fanconi anemia, DNA repair, mitochondria, cancers, inflammation

## Abstract

**Simple Summary:**

Fanconi anemia (FA) is a genetic disorder that is characterized by bone marrow failure (BMF), developmental abnormalities, and predisposition to cancer. In this review, we present an overview of both canonical (regulation of interstrand cross-links repair, ICLs) and noncanonical roles of FA proteins. We divide noncanonical alternative functions in two types: nuclear (outside ICLs such as FA action in replication stress or DSB repair) and cytosolic (such as in mitochondrial quality control or selective autophagy). We further discuss the involvement of FA genes in the predisposition to develop different types of cancers and we examine current DNA damage response-targeted therapies. Finally, we promote an insightful perspective regarding the clinical implication of the cytosolic noncanonical roles of FA proteins in cancer predisposition, suggesting that these alternative roles could be of critical importance for disease progression.

**Abstract:**

Fanconi anemia (FA) is a clinically and genetically heterogeneous disorder characterized by the variable presence of congenital somatic abnormalities, bone marrow failure (BMF), and a predisposition to develop cancer. Monoallelic germline mutations in at least five genes involved in the FA pathway are associated with the development of sporadic hematological and solid malignancies. The key function of the FA pathway is to orchestrate proteins involved in the repair of interstrand cross-links (ICLs), to prevent genomic instability and replication stress. Recently, many studies have highlighted the importance of FA genes in noncanonical pathways, such as mitochondria homeostasis, inflammation, and virophagy, which act, in some cases, independently of DNA repair processes. Thus, primary defects in DNA repair mechanisms of FA patients are typically exacerbated by an impairment of other cytoprotective pathways that contribute to the multifaceted clinical phenotype of this disease. In this review, we summarize recent advances in the understanding of the pathogenesis of FA, with a focus on the cytosolic noncanonical roles of FA genes, discussing how they may contribute to cancer development, thus suggesting opportunities to envisage novel therapeutic approaches.

## 1. Introduction

Fanconi anemia (FA) is a rare genetic disorder caused by an alteration of the genome integrity that affects one in every 100,000 births [1]. It is defined by a very heterogeneous genetic and clinical picture, involving one or more hematopoietic cell lineages and typically characterized by a wide spectrum of congenital somatic abnormalities (including short stature), bone marrow failure (BMF) and the predisposition to develop both hematological and solid malignancies [2]. A short stature mainly results from endocrine defects that about 80% of FA patients show: growth hormone (GH) deficiency, abnormal glucose or insulin metabolism, hypothyroidism, etc. contribute to worsening the life quality of FA patients [3]. Since a considerable overlap exists in the phenotypic manifestations of FA and other genetic disorders and at least 25% of FA patients show few or none of the typical features [4,5], diagnosis may be misdiagnosed or even missed until the onset of BMF or malignancies [6].

FA’s genetic background has been extensively characterized. To date, 22 genes have been identified as mutated in FA (*FANCA*, *FANCB*, *FANCC*, *FANCD1 (BRCA2)*, *FANCD2*, *FANCE*, *FANCF*, *FANCG (XRCC9)*, *FANCI*, *FANCJ (BRIP1* or *BACH1)*, *FANCL*, *FANCM*, *FANCN (PALB2)*, *FANCO (RAD51C)*, *FANCP (SLX4)*, *FANCQ (ERCC4)*, *FANCR (RAD51)*, *FANCS (BRCA1)*, *FANCT (UBE2T)*, *FANCU (XRCC2)*, *FANCV (REV7)*, and *FANCW*) (Table 1). However, since a proportion of otherwise typical FA patients do not present mutations in the known genes, there are probably more genes that have not yet been identified.

## 2. Canonical Role of FA Proteins: FA Signaling in the DNA ICL Repair Pathway

Loss-of-function mutations in any of the FA genes alter the response pathways that repair damaged DNA, specifically interstrand cross-links (ICLs). ICLs are covalent linkage between bases on opposing DNA strands. These highly toxic DNA lesions hamper DNA strand separation upon replication or transcription [7], and, when unresolved, they produce clastogenic effects, leading to genome instability. FA gene-encoded proteins orchestrate the multistep pathway to repair ICLs, known as the FA pathway. Combining nucleotide excision repair (NER) and the highly sensitive homologous recombination (HR) with minor contribution of other types of DNA damage repair (DDR) response, FA genes provide a functional resource to repair ICLs [8]. The canonical mechanism that ensures ICL appropriate repair is tightly regulated. At lesion sites, the interruption of converging replication fork triggers a plethora of events that progressively induce the recruitment of specific FANC proteins [9]. When phosphorylated by the checkpoint ataxia telangiectasia and Rad3-related (ATR) kinase, the helicase FANCM [10], along with Fanconi anemia-associated 24 (FAAP24) and the histone-fold proteins MHF1 and MHF2, localizes at the ICL and acts as a docking site for the FA core complex.

Interestingly, cells depleted for FANCM, FAAP24 or MHF1 are still able to slightly induce FANCD2 monoubiquitination [11]. Accordingly, the E3 ubiquitin ligase UHRF1 has been identified as a novel sensor of ICLs that may substitute for FANCM to some extent to promote FANCD2 recruitment at lesion sites [12,13]. Although the exact function of the FANCM helicase domain remains to be clarified, it has been proposed that the role of FANCM may be stimulatory rather than indispensable. Intriguingly, patients carrying *FANCM* mutation don’t exhibit clinical symptoms that define FA such as congenital malformations and BMF and are just characterized by infertility and cancer predispositions [14].

To ensure proper positioning, FANCS (BRCA1) dislodges the CMG (Cdc45‒MCM‒Gins) helicases complex from the ICL proximity, making the site available for the core complex recognition [15]. The core complex has a modularized and nonredundant activity: it is composed of two modules that arrange the chromatin recruitment, and a catalytic subcomplex. The latter is essential to induce FANCD2‒FANCI heterodimer monoubiquitination [16], promoting the recruitment of the nuclease scaffold protein SLX4 and the endonuclease ERCC1‒ERCC4 heterodimer. This last protein complex is responsible for the “unhooking,” the nucleolytic incision that unties one side of the ICL [17,18], leaving one of the two strands intact, and causing a SSB (single-strand break) on the other.

**Figure 1:**
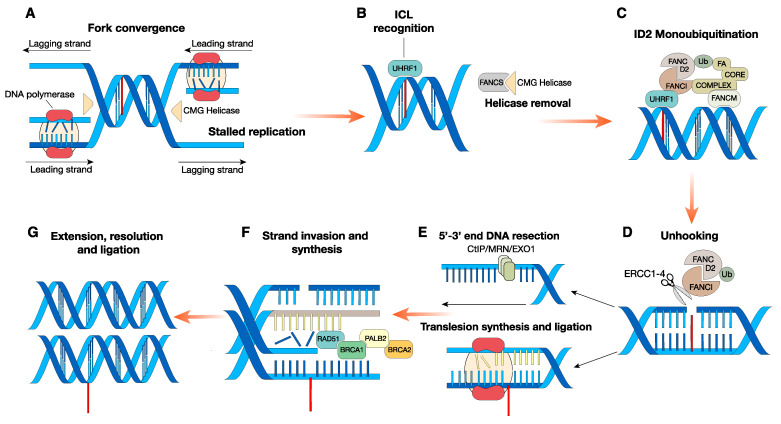
A combination of multiple DNA repair pathways promotes interstrand cross-link repair. (**A**) Converging replication forks stall in proximity of DNA lesion; (**B**) ICL identification by UHRF1. Concurrently, FANCS removes the CMG helicase complex that obstructs the replication fork site, making it available for the FA core complex. (**C**) As a consequence, both the core complex and the FANCD2‒FANCI heterodimer are recruited and the FANCD2‒FANCI complex is monoubiquitinated by the E3 ligase FANCL and its partner, the E2 ligase UBE2T (both members of the FA core complex). (**D**) When ubiquitinated, the ID2 complex promotes the activity of multiple factors such as ERCC1, ERCC4, and SLX4, which coordinate the nucleotide excision responsible for the unhooking. (**E**) At this stage, the two strands have diverging fates: the one that underwent the incision is further processed by nucleases such as CtBP-interacting protein (CtIP), MRN (MRE11–RAD50–NBS1), or exonuclease 1 (EXO1); on the other strand, translation synthesis polymerases bypass the lesion and provide the complementary filament. (**F**) Next, the ssDNA overhang acts as a template for the homologous recombination mediated by RAD51/BRCA2/PALB2/BRCA1 proteins. (**G**) Eventually, after extension and ligation, DNA damage is resolved.

Subsequently, translesion synthesis polymerases provide the intact DNA duplex used as a template for the homologous recombination repair of the damaged DNA strand. Eventually, when activated by BRCA2 and PALB2, the recombinase RAD51 assembles a single-stranded nucleoprotein filament (ssDNA), while the nuclease CtBP-interacting protein (CtIP), MRN, and the exonuclease EXO1 resolve the DSB (double-strand break) and restore the dsDNA [19,20] (Figure 1). ICL agents exist from natural (the most well-known being the by-products of lipid peroxidation such as aldehydes) as well as synthetic sources (chemotherapeutic drugs) [21]. In this regard, despite ICLs inducers being widely employed as drugs for anticancer treatment, the molecular mechanism underlying ICLs recognition and repair has only recently been elucidated. Remarkable insights came from the observation that FA patients’ hematopoietic system is unable to cope with ICLs and displays, as a consequence, harmful susceptibility to ICL inducers.

Homologous recombination (HR) and error-prone classical Non-Homologous End Joining (cNHEJ) have been known as the two subpathways of DSB repair that are more significant for FA pathway-mediated ICL repair. Importantly, cells execute different DNA repair mechanisms depending on the cell-cycle phase they are transiting, with HR confined to S/G2 phases and NHEJ active mainly in the G0/G1 and G2 phases [22]. The HR repair reaction involves three major steps: DSB end resection, strand invasion, and Holliday junction resolution. Consequently, since replication forks activity is distinctive of both S phase and ICLs repair initiation, DDR response is limited to HR, avoiding the potentially dangerous mutations caused by cNHEJ. The complex interplay between HR and FA pathways has been discussed in detail elsewhere [23]. Nevertheless, cNHEJ’s extensive characterization in FA-depleted cells has led to conflicting assumptions as to whether its error-prone activity has detrimental or beneficial effects. Depending on the experimental system and the specific type of DNA damage, the silencing of cNHEJ components in FA pathway-deficient cells can either attenuate or intensify DNA damage. Adamo and colleagues reported that cNHEJ suppression through DNA-PK inhibitors in *Fanca*^−/−^ and *Fancc*^−/−^ MEFs re-established mitomycin C (MMC) sensitivity [24]. In contrast, deletion of the cNHEJ mediators 53BP1 or Ku80 in *Fancd2*-deficient murine B and MEF cells, respectively, exacerbates rather than rescues the sensitivity to the ICL inducers cisplatin and MMC [25].

## 3. Noncanonical Role of FA Proteins

Besides the main role of the FA genes in resolving DNA lesions during replication, many of its factors are involved in other noncanonical processes. Some of these alternative roles are well-characterized and mostly act at the DNA level (outside ICLs removal) to preserve genome stability. In addition to their nuclear localization, more recent cytosolic roles of FA proteins are emerging and may contribute to the disease progression. It is not clear whether these mechanisms are different from the FA pathway or are controlled simultaneously and combined as part of the FA-mediated response. 

### 3.1. Expanded Nuclear Functions of FA Proteins outside ICLs Removal

#### 3.1.1. FA and Replication Stress

In addition to its role in ICL repair, FA proteins also contribute to the maintenance of genome stability by protecting against different replication stressors such as endogenous sources (e.g., oncogenes and aldehydes) or damaging agents (e.g., hydroxyurea) [8]. Intriguingly, some FA proteins protect specific regions of the genome called common fragile sites (CFSs): for example, monoubiquitinated FANCI and FANCD2 maintain the CFSs FRA3B and FRA16D [26], two late-replicating hotspots that reside within large tumor suppressor genes FHIT and WWOX. Moreover, some studies report that the roles of FA proteins depend on the levels of replication stress. Under physiological condition, FANCD2, independently of FANCI, interacts with and recruits the BLM helicase complex to restart stalled replication forks [27]. In addition, FANCD2 and FANCI, independent of the FA pathway, associate with the replicative helicase MCM2–7 complex upon ATR-mediated replication stress [28]. At high levels of replication stress (induced by pharmacological treatment), FANCD2, FANCI, and the FA core complex proteins function synergistically to confer fork stability and promote replication restart [29]. 

Among the plethora of genome-protective mechanism in which FA proteins take part, FANCM and its ability to act as a docking site for both FA core complex and BLM are essential to prevent sister chromatid exchanges (SCEs) and to suppress alternative lengthening of telomeres (ALT). The former typically originates from unresolved D-loops that are not processed through Holliday junction intermediates, while the latter is a telomere maintenance mechanism used by cancers that do not reactivate telomerase expression [30]. Mechanistically, when recruited at lesion site FANCM and BLM complex ensure the dissociation of Rad-51-made D-loops through their intrinsic helicase activity [31,32]. Moreover, it has been demonstrated that FANCM can independently promote branch migration of Holliday junctions, likely impacting on D-loops resolution [33]. FANCM and BLM complex in cooperation with BRCA1 also play an important role in reducing and resolving replication stress that arises spontaneously within ALT telomeres [34]. In detail, Pan and colleagues observed that depletion of FANCM caused induction of telomeric CHK1 signaling, and the recruitment of BLM, RAD51, and BRCA1 at ALT telomeres. In parallel, a most recent study, identified an additional direct effect of FANCM and BLM complex in attenuating ALT activity achieved through their replication fork remodeling capabilities [35]. Both studies exploited the intrinsic predisposition of ALT cancer cells to replication stress by depleting FANCM or through pharmacological inhibition of FANCM-BLM interaction, resulting in synthetic lethality. Another helicase responsible for the maintenance of genome stability is FANCJ. In physiological conditions, guanine-rich sequences form four-stranded structures (G-quadruplexes, G4) that interfere with DNA replication, repair and RNA transcription [36]. To prevent that, FANCJ recognizes G4 through a specific binding motif and mediates multiple rounds of unfolding through its helicase activity [37,38].

#### 3.1.2. FA in DSB Repair

As earlier stated, in FA canonical pathway the DSB caused by the ICLs unhooking is typically repaired via the error-free HR. However, it has been recently elucidated the involvement of several FA proteins in alternative error-prone pathways. A noteworthy case is the FA core complex component FANCA, that, upon FANCG stimulation, catalyzes the bidirectional annealing of single-stranded DNA and strand exchange, playing a direct role in single-strand annealing (SSA) repair pathway [39]. In parallel, despite the ceaseless advances in gene-editing technology, the rationale behind DSB repair pathways in CRISPR-Cas9-mediated mutagenesis is still largely unknown. Nonetheless, recent investigations in Jacob Corn’s laboratory have shed light into the homologous recombination that involves single-stranded template repair (SSTR) programmed from synthetic single-stranded oligodeoxyribonucleotides (ssODNs). Exploiting a novel coupled inhibition-cutting system based on a CRISPRi library, they observed a robust deletion of genes annotated in the FA pathway in cell populations where gRNAs target genes required for SSTR. Additionally, they also discovered that FA pathway promotes SSTR at the expense of NHEJ, minimizing the effects of error- prone end-joining pathways [40].

#### 3.1.3. FA and Transcription‒Replication Conflicts

The relevance and frequency of both DNA replication and transcription during the cell life cycle facilitate the encounter of the machineries responsible for these two processes. Collisions between replication forks and the elongating RNA polymerases represent a source of genome instability, a hallmark of cancer development. These conflicts may also be caused by indirect structures originating from the transcriptional activity itself. Therefore, cells have evolved different strategies to prevent their accumulation [41]. Among the transcription by-products that hinder replication forks progress, R-loops are major inducers of genome instability. R-loops arise from the combination of RNA‒DNA hybrids with a single-strand DNA, and recent advances have established that the FA proteins are involved in their removal at multiple stages [42]. The observation that BRCA2 cooperates with the TREX-2 complex, a mRNP biogenesis and export regulator, to process R-loops and prevent the related genomic instability [43], has paved the way for the unveiling of a profound interplay between FA proteins and R-loops clearance. Taking advantage of a genome-wide analysis, Hatchi and colleagues showed that FANCD2 and its binding partner senataxin (SETX) are also enlisted at R-loop-rich termination regions of highly transcribed genes [44]. Moreover, FANCM branch migration activity is crucial to resolve RNA‒DNA hybrids promoting replication fork restart and avoiding accumulation of DNA damage [45]. Accordingly, a recent genome-wide trigenic interaction screening evaluated critical genes for genome fitness and survival in the absence of RNaseH (typically responsible for RNA‒DNA hybrid processing) and identified the MRN complex as pivotal for R-loop suppression. Mechanistically, the MRN complex acts as a scaffold for the recruitment of FANCM upon R-loops. [46]. Collectively, these data reveal a more comprehensive role for FA proteins in genome integrity maintenance.

Accumulating evidence indicates that subsets of FA proteins also participate in regulating cell division to safeguard chromosomes during mitosis (reviewed in detail by [47]). For example, some FA proteins (FANCA-B-C-E-G-L, BRCA2, FANCD2, and PALB2) localize to centrosomes to preserve the mitotic machinery [48], or at the mitotic exit, some FA proteins, such as BRCA2, recruit cytokinesis effectors to control cytokinesis [49].

### 3.2. Emerging Cytosolic Roles of FA Proteins

**Figure 2:**
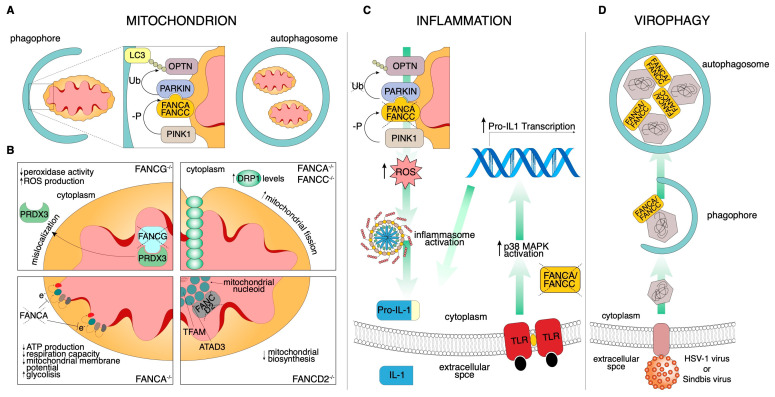
Schematic of the noncanonical FA pathways. (**A**) During mitophagy, damaged mitochondria are selectively engulfed by the phagophore. To properly identify the substrate, the E3 ligase PARKIN ubiquitinates the mitophagy receptor, which acts as an adapter for LC3-mediated autophagosome recognition. In this context, FANCC assists PARKIN localization to the mitochondria. (**B**) FA protein-related mitochondrial functions. (Top left) FANCG-deficient cells display PRDX3 mislocalization. The consequent reduced mitochondrial peroxidase activity increases ROS production. (Top right) FANCA and FANCC downregulated cells show increased DRP1 levels resulting in an increment of mitochondrial fission. (Bottom left) FANCA deficiency impacts negatively on mitochondrial electron transport chain efficiency, reducing ATP production and respiration capacity and altering the mitochondrial membrane potential. This inevitably leads to a shift from aerobic to glycolytic metabolism. (Bottom right) FANCD2 mitochondrial nucleoid complex interaction regulates mitochondrial biosynthesis. (**C**) FANCA and FANCC knockdown increase the pro-inflammatory secretion of IL-1β in a dual convergent manner. On the left, impaired mitophagy stimulates inflammasome activation through ROS overproduction and, consequently, IL-1β is secreted. On the right, exogenously stimulated Toll-like receptors activate a signal transduction cascade that culminates with Pro-IL-1β transcription. (**D**) Both FANCA and FANCC are required for Sindbis and HSV-1 nucleocapsid recognition to control viral infection by virophagy.

Although major clinical conditions in FA result from defects in DNA damage and repair machinery, recent discoveries suggest that other cellular pathways are also compromised and contribute to the disease progression (Table 1). It has long been appreciated, indeed, that some *FA* genes are involved in additional cytoprotective pathways (Figure 2), such as defense from reactive oxygen species (ROS)-induced cell death [50], mitochondrial homeostasis [51], and defense from pro-inflammatory cytokine-induced apoptosis [52]. Intriguingly, many of the noncanonical functions of FA proteins discussed below are not restricted to HSCs and some of these processes may be independent of the DNA damage response (DDR).

#### 3.2.1. Mitochondria

Defective mitochondria are one of the sources of pro-inflammatory signaling pathways, through the production of ROS and an associated increment of oxidative stress [53]. A large amount of research has been dedicated to uncovering how FA signaling affects mitochondrial functions. 

Oxidative stress: Oxidative stress is generally defined as an imbalance that favors the production of ROS over antioxidant defenses; the majority of ROS are produced by mitochondrial respiration. FA cells (e.g., *FANCA* mutant fibroblasts) have impaired electron transport in Complexes I and III, leading to changes in the relative ATP/AMP ratio, which results in a decreased respiration capacity, mitochondrial membrane potential, and oxygen uptake [54,55,56,57,58]. This is also supplemented by inactivation of essential enzymes of the energy production pathway. From a molecular point of view, FANCA, FANCC, and FANCG are found to interact with cytochrome P450-redox related activities and to respond to oxidative damage [58,59]. In addition, Mukhopadhyay and colleagues identified FANCG protein in mitochondria, as well as its interaction with the mitochondrial peroxidase peroxiredoxin3 (PRDX3) [60]. In FA cells, however, PRDX3 is mislocalized and thioredoxin-dependent peroxidase activity strongly deregulated. More recently, a mitochondrial localization signal (MLS) on FANCG has been identified; in eight FA patients, indeed, a single nucleotide change (C.65G>C) leads to the conversion of the amino acid arginine at the 22 positions of the MLS into proline (p.Arg22Pro) [61]. This mutant protein (R22P) fails to localize to the mitochondria and protect them from oxidative stress; on the contrary, this mutant is still able to participate to the formation of the FA core complex in the nucleus and is also resistant to ICL agents. More interestingly, in R22P stable cells, there is also an iron deficiency of FA protein FANCJ, an iron‒sulfur (Fe‒S)-containing helicase involved in DNA repair [61]. This suggests, for the first time, that oxidative stress-mediated mitochondrial dysfunction causes, per se, defective FANCJ, leading to genomic instability.

Metabolism: Reflecting the crucial role of the mitochondria in aerobic ATP production, normal metabolism is altered in FA cells and probably complementary pathways are involved in prevailing the energetic defect. In the first systematic work about FA metabolism, glycolysis emerged as the main source when aerobic metabolism was reduced by unproductive mitochondrial electron transport complexes [62]. However, glycolysis remains insufficient to satisfy FA cells’ energy requirements. Since energetic metabolism plays an essential role during HSCs’ differentiation into lymphocytes, this could, at least in part, explain a defective metabolic maturation in the bone marrow during the exit from the homeostatic quiescent state.

Morphology: Oxidative phosphorylation (OXPHOS) impairment is not the only mitochondrial damage in FA cells. Several reports demonstrate alterations in mitochondrial morphology; mitochondria appear swollen with matrix rarefaction, altered cristae, and reticulum fragmentation. For example, mitochondria in FANCD2 mutant cells show wall ruptures, thinner walls and cristae, and smaller sizes [63], as well as mitochondria from *FANCG*^−/−^ fibroblasts displaying frequent elongation and irregular shapes [60]. Two opposing coordinated processes, fusion and fission, determine mitochondrial content and structure and are essential for maintaining ordinary mitochondrial function and regulating mitochondrial morphology [64]. Mitochondrial fission involves the recruitment of GTPase dynamin-related protein (DRP1) from the cytosol to the mitochondrial membranes to catalyze the fission reaction [65]. Shyamsunder and colleagues found an accumulation of DRP1 in the mitochondria of FANCA and FANCC-deficient cells, which positively affects mitochondria fission [66]. 

Mitophagy: A specific cellular process called mitophagy removes damaged and old mitochondria through double-membraned vesicles known as autophagosomes. In contrast to bulk autophagy, which removes parts of the cytoplasm nonspecifically, mitophagy is one of the forms of selective autophagy that precisely removes unnecessary cytoplasmic contents (e.g., bacteria, mitochondria, and endoplasmic reticulum) [67]. In 2016, Sumpter and colleagues, using a CRISPR/Cas9-mediated approach in HeLa cells, FANCC mutant fibroblasts of FA patients, and bone marrow-derived macrophages from *Fancc*-deficient mice, described the role of FANCC, as well as of other FA genes, in mitophagy [68]. Moreover, through siRNA experiments, FANCF and FANCL have also been found to be required in mitophagy, providing a novel role for these FA proteins. In detail, FANCC is recruited to the mitochondria and interacts with the E3 ligase PARKIN, a key enzyme that regulates mitochondrial degradation by mitophagy in a mitochondrial damage-dependent manner. *FANCC*-deficient cells, indeed, show accumulation of damaged mitochondria, suggesting a defect in the mitophagy process [68]. This is in line with a previous study that found mitochondrial fission as a precondition for mitophagy defects in FA. The blockade of this process may allow autophagy to remove dysfunctional mitochondria [66]. Interestingly, the function of FANCC and FANCA in mitophagy seems to be genetically separate from their role in DDR [69]. Indeed, the hypomorphic mutants of FANCC (c.67delG) and FANCA (p.Arg951Gln/Trp, p.His913Pro) are not functional in DNA repair but preserve the mitochondria functions. Patients with these mutations have a mild clinical disease, suggesting the importance of FA-mediated mitochondria removal in improving the disease course. 

Biosynthesis: Homeostasis of mitochondrial mass is maintained by a balance between mitophagy and mitochondrial biogenesis. A set of DNA-binding core proteins involved in mtDNA maintenance and transcription forms the mitochondrial nucleoid. The most frequently identified components of this complex, essential for mitochondrion biosynthesis, are ATAD3 and translation mitochondrial factor of elongation (TUFM). ATAD3 is an ATPase that plays a central role in nucleoid organization, as it associates with both the inner membrane and the mitochondrial ribosome, and also binds to D-loop sequences of mtDNA [70]. Intriguingly, by a proteomic approach, FANCD2 has been found to be associated with both Atad3 and Tufm and, through its localization at the mitochondrion, regulates Atad3/Tufm expression [71], thus providing the first evidence for FANCD2 as a crucial player of mitochondrial biosynthesis. Intriguingly, by a proteomic approach, FANCD2 has been found to be associated with the nucleoid complex components Atad3 and Tufm and, through its localization at the mitochondrion, regulates Atad3/Tufm expression [71], providing the first evidence for FANCD2 as a crucial player of mitochondrial biosynthesis.

#### 3.2.2. Endocrinopathies

About 80% of FA children and adults show at least one endocrine defect, including GH deficiency, atypical glucose or insulin metabolism, dyslipidemia, hypothyroidism, pubertal delay, hypogonadism, or impaired fertility. Very recently, the first genotype‒phenotype correlation of FA endocrine defects has been studied in a cohort of 24 patients homozygous for a founder mutation (c.637_643del (p.Tyr213Lysfs*6)) in *FANCG* [72]. Defects usually observed in FA patients are related to glucose and insulin metabolism as well as to lipid dysregulation [73]. Mice deficient for the *Fanca* or *Fancc* gene seem to be diabetes- and obesity-prone [74]. In this study, insulin resistance has been associated with high levels of ROS that characterized several FA mice tissues such as liver, fat, and muscles. Other reports underline a role for iron overload in heavily FA transfused patients, or the use of androgens and corticosteroids during FA treatment [3]. With respect to lipid metabolism, using MS-based lipidomics approaches, Zhao and colleagues demonstrate that FA DNA repair loss is correlated with a defined lipid signature, opening up a new scenario for potential diagnostic implications [75].

#### 3.2.3. Inflammation 

One of the features of FA cells is the increase in inflammation markers and hypersensitivity to pro-inflammatory cytokine-induced apoptosis such as TNF-α [76,77,78,79], IL-6 [80], and IL-1β [81]. Of course, persistent DNA damage is well known to excite the production of inflammatory mediators [82,83], which probably impact on bone marrow activity. Activation of toll-like receptors in FANCA and FANCC-deficient monocytes abnormally increases IL-1β expression, which negatively affects HSCs’ self-replication [52]. Interestingly, hampering the action of the inflammatory cytokine interleukin 1 beta (IL-1β), using IL-1 receptor blockade, avoids bone marrow failure [84]. However, how DNA damage plays a role in this pathway is not well understood. Of course, parallel noncanonical pathways could contribute to the progression of BMF in FA through ROS production. In *FANCC*^−/−^ primary bone-marrow-derived macrophages (BMDMs), indeed, an impaired bacterial lipopolysaccharide (LPS)-mitophagy is associated with ROS-dependent inflammasome hyperactivation and higher IL-1β secretion, resulting in the persistence of pathogen-associated and danger associated molecular patterns (PAMPs and DAMPs) [68]. Moreover, a range of intricate secondary effects likely account for some of the inflammatory susceptibilities of FA HSCs. Using isogenic cells derived from patients and from nullizygous mice carrying inactivating mutations in the *FANCC* gene, Pang and colleagues discovered that FANCC protects against proinflammatory cytokine-induced cell death by interacting with signal transducer and activator of transcription 1 (STAT1) [85] and stress-inducible heat shock protein 70 (HSPA1A) [86]. Mutations in the *FANCA*, *FANCC*, and *FANCG* genes, indeed, markedly increase the interaction between eukaryotic translation initiation factor 2-alpha kinase 2 (PKR) and FANCC, leading to the hypersensitivity of BM progenitor cells to growth repression mediated by IFN-γ and TNF-γ [87]. However, further studies are necessary to confirm that these effects are truly independent of induced DNA damage.

#### 3.2.4. Virophagy

Autophagy is also one of the most ancient cell-autonomous protection mechanisms to contrast pathogen invasion. Viral components can be engulfed and directed to autophagosomes for degradation through a process named virophagy [88]. By high-content genome-wide screening, three FA genes, *FANCC*, *FANCF*, and *FANCL*, have been found to be required for virophagy. Importantly, FANCC (as well as FANCA) binds the Sindbis virus capsid protein, mediating its degradation; analyzing *FANCC*^−/−^ cells, a defect in the virophagy of a herpes simplex virus type 1 (HSV-1) was also found [50]. In light of these findings, Sumpter and colleagues posited a key role for FANCC in antiviral host defense, with *FANCC*^−/−^ cells being more susceptible to lethal central nervous system (CNS) infections.

## 4. Hematopoietic Defects in FA Patients

As mentioned above, the peculiar alteration of FA pathway impacts negatively on the hematopoietic system. The reasons for this susceptibility have not yet been fully revealed, but the evidence argues for a strong connection between the FA pathway and hematopoietic stem cells’ (HSCs) expansion and quiescence. Indeed, HSCs’ functionalities are severely affected by DNA damage repair impairment in stressful conditions. Accordingly, FA patients display a significant reduction in CD34^+^ cells, indicating HSCs’ pool depletion [89]. Moreover, the different transient HSC states are related to sensitivity to genomic instability. Quiescent populations activate NHEJ in response to ionizing radiation, contributing to the acquisition of genomic rearrangements and consequent hematopoietic abnormalities [90]. In patient-derived HSCs, instead, the cell cycle is halted in the G0/G1 phase in a p53/p21-dependent manner [91], disturbing their expansion and inevitably reducing their differentiated subpopulations [92]. Further insights into FA HSCs biology came from an elegant study of Zhang and colleagues, which demonstrates that the hyperactivation of TFG-β pathway is a distinctive feature of BMF and its inhibition in hematopoietic stem and progenitor cells (HSPCs) may promote HR repair of DSB with a concomitant reduction in NHEJ [93].

The essential ability of stem cells to preserve their plasticity also requires unique metabolic features. Based on this, many groups focused their efforts on elucidating how reactive molecules, capable of inducing DNA damage, contribute to aggravate FA patients’ condition. It is well established that the low oxygen tension generated by hypoxic conditions favors FA-deficient cells and iPSC lines’ [94] survival, implying a prominent role of ROS production in their viability. However, using a transgenic mouse model, Hadjur and colleagues demonstrated that the double impairment of both the FA pathway and superoxide dismutase (SOD1), an enzyme required for ROS metabolism (*Fancc*^−/−^ *Sod1*^−/−^), weakly affects both HSCs and bone marrow [95]. 

The FA genes’ involvement in ICLs repair also implies a connection with endogenously produced aldehydes. To avoid the accumulation of these alkylating agents, *Aldh2*, a mitochondrial aldehyde dehydrogenase, converts acetaldehyde to carboxylic acid. When *Aldh2* and *Fancd2* are simultaneously disrupted, this produces a synergistic detrimental effect that results in embryonic lethality. The inability to conclude successfully the gestation is reverted when the aldehyde catabolism of the mother is still active as in *Aldh2* heterozygous condition. When born, *Aldh2*^−/−^ *Fancd2*^−/−^ mice display HSCs compromised functionality and, as a consequence, BMF [96]. The effect of *ALDH2* mutation in FA patients has been further analyzed in Japanese population, where a remarkable fraction (~50%) carries Glu504Lys substitution (hereafter referred as allele A), a mutation that strongly impairs aldehydes catabolism. In contrast with the aforementioned murine model, recent examination of a cohort of Japanese FA infants revealed that none of the distinctive clinical parameters are influenced by maternal *ALDH2* genotype [97]. Otherwise, the FA patient’s phenotype is affected by their own ALDH2 activity, as patients with *ALDH2* AA genotype display earlier onset of BMF and a relatively more severe clinical picture [98,99]. These data strengthen the evident correlation between FA and the endogenous aldehyde catabolism.

Investigation of the hematological abnormalities in mouse models is difficult for many technical reasons. First of all, deletion of *Fancd1*, *Fancn*, *Fanco*, *Fancc*, *Fancd2*, *Fancl*, *Fancm*, and *Fancp* is embryonically or perinatally lethal. The remainder not only exhibits reduced fertility but also few developmental abnormalities. Secondly, and most importantly, only hypomorphic *Fancd1*, *Fancd2*, and *Fancp* mice display the peculiar anemia of FA patients or HSCs underrepresentation, while the others are unaffected [100].

Taken together, these data support a scenario in which HSCs’ metabolism and their DNA damage response are strictly linked to the clinical features of FA.

## 5. FA Proteins and Cancer Predisposition

The identification of the relevant role of *BRCA1* and *BRCA2* tumor suppressor genes [101,102,103,104] in FA pathogenesis [105,106] has solidly linked FA genetic background to DNA repair defect, and hence to cancer predisposition. 

Germline monoallelic mutations or promoter hypermethylation of FA genes sporadically increase cancer susceptibility in the non-FA population, suggesting a gene dosage effect in ICL repair activity. Apart from the well-established role of *BRCA1* and *BRCA2* in breast cancer development, as well as in ovarian, pancreatic, prostate, and stomach tumors [107], germline monoallelic mutations in other FA genes have been implicated in increased risk of multiple cancer types. Mutations in *PALB2/FANCN* increase breast and pancreatic cancer incidence, while truncating variants of *BRIP1/FANCJ* and *RAD51C/FANCO* increase ovarian [108,109] but not breast cancer [110]. Moreover, exome sequencing studies highlighted that *FANCC* and *FANCM* also confer susceptibility to breast cancer [111,112], even if the evidence presented did not reach the statistical significance proposed for cancer mutated in DNA-damage genes [113]. As mentioned above, *FANCM* loss of function causes early-onset cancers but not FA [114]. In these studies, some individuals carrying biallelic nonsense mutations in *FANCM* were identified: a patient shows B-cell precursor lymphoblastic leukemia, two siblings developed squamous carcinomas in the mouth and neck and five cases are correlated with breast cancers. [115]. Interestingly, *Fancm* knockout mice also have an increased incidence of cancers [116].

In 2015, Zhang and colleagues reported the results of a next-generation sequencing analysis on constitutional DNA from 1120 children with a variety of malignancies, aimed at defining the prevalence of germline mutations predisposing to childhood cancer [117]. Germline heterozygous mutations in *BRCA1*, *BRCA2*, and *PALB2* were found in eight children with a spectrum of cancers including leukemia, CNS tumors, neuroblastoma, osteosarcoma, and rhabdomyosarcoma (Table 1). Although some of these results have not reached population-level statistical significance, the investigation of new genes and mutations is vital to improve treatment outcomes and patient survival. Recent studies reported a 30% chance to develop cancer in FA patients, regardless of the mutated gene [118]. Biallelic mutations in *BRCA2* and *PALB2* predispose FA patients to develop acute myelogenous leukemia (AML) and embryonic tumors, such as medulloblastoma, neuroblastoma, and Wilms tumors, while mutations in other FA genes are associated with an increased incidence of squamous cell carcinoma, mostly affecting the head and neck (HNSCCs) and vulvovaginal regions. This suggests that predisposition to a specific form of cancer could depend on the biallelic mutated gene. The onset of cancers, such as AML and HNSCCs, takes place at an unusually early age in FA individuals when compared to the general population. The transformation may arise through similar or different mechanisms and it has been hypothesized that FA proteins could commonly regulate some cellular processes and others in a tissue-exclusive manner [119]. Published works suggest that BMF could force compensatory chronic proliferation, resulting in clonal evolution and AML [120]. Recent findings suggest that germline heterozygous rare and novel *FANC* gene variants impair the FA DNA repair pathway in HSC, resulting in a reduced capacity to preserve genome integrity, which may in turn contribute to an increased risk of AML [121]. Intriguingly, HNSCC is the most frequently diagnosed solid tumor in FA patients, even if no specific gene has been identified as related with this susceptibility. The reasons for the 500‒700-fold increased incidence of squamous cell carcinoma are still up for debate. Without risk factors (such as tobacco exposure), indeed, the increment in the genomic instability in the epithelial cells of the head and neck region could play a role [121], or the reduced total NK cells may unfavorably impact on the immune surveillance against cancer cells [122] and/or create increased susceptibility to human papillomavirus (HPV)-induced carcinogenesis; however, these associations remain putative and need further investigations [123]. Additionally, a TCGA computational analysis demonstrated that somatic genetic alteration of FA genes are widely diffused in multiple cancer types [8].

The FA proteins are the main players in the maintenance of genome stability through DNA damage repair, replication fork stabilization, and mitotic stress mitigation. Genome instability is defined as an acquired state that allows for an increased rate of spontaneous genetic mutations throughout each replicative cell cycle and represents a hallmark of cancer. The complex interplay between FA genes, genome instability, and cancer has been discussed in detail elsewhere (see [47,124,125,126]).

### 5.1. DNA Damage Response-Targeted Therapies in FA Patients

Interstrand cross-linking agents are typically used in anticancer therapy for patients with somatic mutations in FA genes; however, they are extremely deleterious in patients harboring germline biallelic mutations in the same genes. In these patients, indeed, interstrand cross-linking agents (such as platins, cyclophosphamide, and mitomycin C)-induced mitotic catastrophe and p53-dependent apoptosis contribute to a severe, prolonged, or even irreversible myelosuppression due to genetic instability [127,128]. Moreover, both the intrinsic defect in DNA repair and hypersensitivity to cytotoxic agents of FA patients further complicate the use of allogeneic hematopoietic stem cell transplantation (HSCT); this currently represents the only option with the potential for definitively correcting bone marrow failure, as well as preventing/treating the hematopoietic malignancies associated with the disease. In FA patients, initial attempts at HSCT had poor results, mainly due to excessive toxicity and severe acute graft-versus-host disease (GvHD) occurring after chemotherapy and radiation, which are used in standard pretransplantation conditioning regimens [129,130]. This has led to the development of specific protocols for FA patients, based on the use of fludarabine, low-dose cyclophosphamide and irradiation, which improved transplantation outcomes [131,132]. Despite the reduction in early transplantation-related mortality, a significant proportion of HSCT-treated FA patients develop squamous cell carcinoma, especially after chronic GvHD. Preliminary studies, however, positively evaluated TFG-β inhibition by neutralizing antibodies in FA mice laying the ground for a novel approach to treat BMF in FA patients [93].

Currently, curative cancer therapies exploit the phenomenon of “oncogene addiction” to synthetically target pathways upon which cancer cells rely for their proliferation and/or survival. In this context, HR-defective cells have been shown to be extremely susceptible to poly(ADP-ribose) polymerase (PARP) inhibition, with relatively nontoxic effects on normal cells [133]. In standard conditions, PARP activity is important to facilitate SSB and nucleotide excision repair. When PARP is inhibited, cells are compelled to depend on HR to cope with genomic instability [134,135]. Taking advantage of this mechanism, many clinical trials evaluated PARP inhibitors’ effects in *BRCA1*/*2*-mutated breast or ovarian cancers with positive results [136,137]. Moreover, the first FDA-approved PARP inhibitor (olaparib) has shown promising effects in the treatment of patients with germline mutations in both *BRCA1* and *BRCA2* [138,139]. Currently, a phase III clinical trial (NCT01945775) for advanced and/or metastatic breast cancer patients with *BRCA1*/*2* deleterious mutations is testing another PARP inhibitor (BMN673) that shows the highest trapping activity on its substrate. Intriguingly, despite the lack of a molecular pathway characterization, a recent study demonstrated the promising potential efficacy of two approved epidermal growth factor receptor (EGFR) inhibitors for FA-related head and neck squamous cell carcinoma [140].

### 5.2. Clinical Implications for Emerging FA Noncanonical Roles in Cancer Predisposition 

Besides being critical to some nuclear functions such as DDR of ICLs, the FA proteins also have emerging roles in other nuclear and cytoplasmic functions, opening up innovative therapeutic options. The onset and progress of BMF in FA patients, indeed, is clinically variable and the molecular mechanisms are poorly understood. The previously discussed findings suggest that noncanonical roles may be a point of convergence in the pathological manifestations of FA. Nonetheless, whether these processes are independent of the DNA repair function, being upstream events that contribute to DNA damage, or a consequence of it, is currently unclear. However, extensive evidence exists to support the idea that HSCs dysfunction is exacerbated or activated by the effect that FA mutations have on noncanonical processes, since these pathways play a fundamental role in protecting cells. Moreover, some of the noncanonical roles of FA proteins described below would not be restricted to HSCs. It is plausible to suppose that the multifunctionality of some of the FA proteins also has a potential role in FA cancer predisposition, probably contributing to the selection of neoplastic clones. In this section, we discuss possible connections between emerging cytosolic roles of FA proteins and cancer predisposition.

Mitochondria are crucial for all aspects of tumor progression, being involved not only as ROS producers, but also for regulating cell metabolism and inflammation [141]. Furthermore, evidence indicates that dysfunction of mitophagy promotes tumorigenesis and neoplastic progression [142,143], even though the way they are connected is strictly dependent on the cancer type. This assumption is supported by several examples where the mutation/downregulation of some mitophagy receptors (proteins that link damaged mitochondria to autophagosome) such as BCL2/adenovirus E1B 19 kDa protein-interacting protein 3 (BNIP3) or mitophagy-related enzymes (such as PTEN-induced putative kinase 1 (PINK1) or PARKIN) has been identified in many human cancer types [144]. We can speculate that, upon solid tumor onset, a defect in hypoxia-induced mitophagy could prevent cell death and support cancer progression. In the context of FA, we believe that the evaluation of mitochondria alterations could open up new horizons for identifying novel therapeutic targets against FA-related diseases. Very recently, molecular insights have emerged about the role of FANCS (BRCA1) in regulating the mitophagy process; FANCS, indeed, inhibits ataxia-telangiectasia mutated (ATM)-AMP-activated protein kinase (AMPK)-DRP1-mediated mitochondrial fission and promotes tumor proliferation and invasion [145]. In addition, defects in mitophagy are emerging as a common feature of a variety of cancer predisposition syndromes (e.g., Xeroderma pigmentosum and Ataxia telangiectasia), suggesting that treatments to increase mitophagy (by helping to remove the main endogenous source of ROS) could improve patient outcomes [146,147]. A similar consideration could justify the use of antioxidants to decrease cancer incidence in FA. Studies in *Fancd2*^−/−^ mice have demonstrated that long-term administration of tempol and resveratrol decreases cancer incidence and hematopoietic defects [148,149]. Interestingly, resveratrol is a potent autophagy inducer that has recently been found to ameliorate mitophagy disturbance [150], suggesting that the stimulation of mitochondrial turnover could be a promising approach to decrease FA cancer predisposition. 

Concerning the endocrine defects observed in FA patients, it is not known whether and how FA genes’ deficiency may contribute to the beginning of obesity, diabetes, and/or inflammation, which are known to be strictly connected to cancer [151]. In 2016, it was reported that FAVL, a novel splice variant of *FANCL*, impairs the FA pathway and contributes to bladder cancer development at the metabolic level. The same authors found that, in cells expressing FANCC at different levels, there are alterations in metabolites associated with aging, inflammation, and cancers [152]. Moreover, Zhao and colleagues, analyzing lipid profiling in FA-isogenic HNSCC keratinocyte cell lines, found that the increase of glycosphingolipids such as the ganglioside GM3 fuels FA-deficient HNSCC cells’ invasive proprieties [75].

About the susceptibility to viral infection-mediated carcinogenesis, many studies of HNSCC, anogenital warts, and other SCCs in individuals with FA have suggested a role for HPV in FA-associated cancers. Although the role of HPV in FA-related head and neck tumors remains controversial [153,154], different hypotheses have emerged about HPV susceptibility or persistence. On the one hand, the immune impairment associated with FA could play a role [155], and the prolonged state of immunosuppression and late immune reconstitution may predispose FA patients to viral infections and the consequent cellular changes involved in tumorigenesis. On the other hand, defects in virophagy cannot be ignored. During viral infections, autophagy is an essential response that could guarantee virus depletion and cell survival [156]. In *Papillomavirus* infection, HPV has been reported to inhibit autophagy, thus both avoiding early viral degradation during de novo infection and promoting the malignant transformation of infected cells [157]. Although more studies are necessary to determine the connection between virophagy, HPV infection, and increased head and neck carcinomas in individuals with FA, vaccination of these patients is highly recommended to prevent early viral infection and subsequent virus-induced squamous cell carcinoma. 

A fundamental connection among defects in selective autophagy (mitophagy/virophagy), in mitochondrial quality control, and in preventing inflammation is supported by the presence of increased mtROS in FA-deficient cells [68]. Dysregulated mtROS production is sufficient to induce metabolic reprograming of cancer cells, permitting an adaptation to oxidative stress that ultimately advantages tumorigenesis and chemoresistance [158]. We speculate that increased mtROS could alter homeostatic functions acting as new drivers of tumorigenesis in the setting of FA gene defects/mutations.

## 6. Conclusions

Cancer predisposition is a typical genetic susceptibility beyond anemia and bone marrow failure in FA patients with biallelic modifications in FA genes. The FA protein network provides multifaceted genome guardianship extending beyond the repair of DNA ICLs. Although the precise mechanism(s) by which FA proteins act in noncanonical processes and how this contributes to FA-associated cancers remain to be determined, a deeper knowledge of how FA noncanonical alterations contribute to tumor transformation and progression could open up new horizons for the diagnosis and therapy of neoplasia in FA patients.

## Figures and Tables

**Table 1:** Canonical and noncanonical roles of FA proteins.

FA Gene	Alternative Name and Location		Key Features	Canonical Role	Cytosolic Noncanonical Role	Cancer Type Predisposition
*FANCA*	-	16q24.3	Fanconi anemia core complex	Promotes FANCD2/I heterodimer monoubiquitylation	Interact with cytochrome P450 to respond oxidative damage; mitophagy effector	-
*FANCB*	-	Xp22.2	Fanconi anemia core complex	Promotes FANCD2/I heterodimer monoubiquitylation	-	-
*FANCC*	-	9q22.32	Fanconi anemia core complex	Promotes FANCD2/I heterodimer monoubiquitylation	Interact with cytochrome P450 to respond oxidative damage; mitophagy effector through PARKIN interaction; virophagy mediator	Breast cancer
*FANCD1*	BRCA2	13q12. 3	RAD51 activator	Fork stabilization; homologous recombination	Involved in mitophagy	AML; T-ALL; ALL; brain tumor; medulloblastoma; squamous cell carcinoma; breast cancer
*FANCD2*	-	3p25.3	FANCI binding partner	Monoubiquitylated by the core complex; promote the unhooking	Regulation of mitochondrial nucleoid complex components Atad3 and Tufm; sustain stem cells functions through SF3Bq spliceosomal protein interaction; Involved in mitophagy	-
*FANCE*	-	6p21.31	Fanconi anemia core complex	Promotes FANCD2/I heterodimer monoubiquitylation	-	-
*FANCF*	-	11p14.3	Fanconi anemia core complex	Promotes FANCD2/I heterodimer monoubiquitylation	Involved in mitophagy; virophagy mediator	-
*FANCG*	-	9p13.3	Fanconi anemia core complex	Promotes FANCD2/I heterodimer monoubiquitylation	Interact with cytochrome P450 to respond oxidative damage; involved in mitochondria morphology regulation	-
*FANCI*	-	15q26.1	FANCD2 binding partner	Several functions in the ICL repair	Sustain stem cells functions through SF3Bq spliceosomal protein interaction	-
*FANCJ*	BRIP1	17q23.2	BRCA1-interacting protein helicase	Promotes HR through BRCA1 binding	DNA-dependent ATPase activity	Ovarian cancer
*FANCL*	-	2p16.1	E3 ubiquitin ligase of the Fanconi anemia core complex	Promotes FANCD2/I heterodimer monoubiquitylation	β-Catenin ubiquitination; involved in mitophagy; virophagy mediator	-
*FANCM*	-	14q21. 2	ATR-activated DNA helicase	Recruits the FA core complex	-	Breast cancer
*FANCN*	PALB2	16p12.2	Partner and localizer of BRCA2	Promotes HR and RAD51 activity	-	AML; medulloblastoma; neuroblastoma; Wilms Tumor; breast cancer
*FANCO*	RAD51C	17q23	RAD51 paralog	HR	-	Ovarian cancer
*FANCP*	SLX4	16p13.3	Multidomain scaffold protein	Nuclease scaffold for ICL unhooking	-	
*FANCQ*	ERCC4	16p13.12	DNA repair endonuclease	DNA incision for ICL unhooking	-	
*FANCR*	RAD51	15q15.1	DNA recombinase	Fork stabilization and HR	Regulation of axonal branching through Netrin-1 modulation	
*FANCS*	BRCA1	17q21.31	E3 ubiquitin ligase	CMG complex unloading; Homologous recombination	Involved in mitophagy	Breast, ovarian cancer and leukemia
*FANCT*	UBE2T	1q32.1	E2 ubiquitin ligase of the Fanconi anemia core complex	Promotes FANCD2/I heterodimer monoubiquitylation	-	
*FANCU*	XRCC2	7q36.1	DNA repair protein	HR	-	
*FANCV*	REV7	1p36.22	DNA polymerase	Translesion DNA synthesis	-	
*FANCW*	-	16q23.1	E3 ubiquitin ligase	Essential for RAD51 turnover	-	
